# Integrated Analysis of ECT2 and COL17A1 as Potential Biomarkers for Pancreatic Cancer

**DOI:** 10.1155/2022/9453549

**Published:** 2022-06-08

**Authors:** Wen-liang Huang, Shu-fen Wu, Xiao Huang, Shan Zhou

**Affiliations:** ^1^MRI Room, The Second Affiliated Hospital of Luohe Medical College, Luohe, China; ^2^Department of Gynecology and Obstetrics, The Second Affiliated Hospital of Luohe Medical College, Luohe, China; ^3^Department of Clinical Laboratory, Zhengzhou University First Affiliated Hospital, Zhengzhou, China

## Abstract

**Background:**

Pancreatic cancer (PC) is a malignant tumor of the digestive tract. It presents with atypical clinical symptoms and lacks specific diagnostic indicators. This study is aimed at exploring the potential biomarkers of PC.

**Methods:**

TCGA database pancreatic cancer dataset was normalized and used to identify differentially expressed genes (DEGs). Survival, independent prognostic, and clinical correlation analyses were performed on DEGs to screen for key genes. DNA methylation, mutation, and copy number variation (CNV) analyses were used to analyze genetic variants in key genes. GSEA was performed to explore the functional enrichment of the key genes. Based on the expression of key genes, construction of a competing endogenous RNA (ceRNA) network, analysis of the tumor microenvironment (TME), and prediction of chemotherapeutic drug sensitivity were performed. Furthermore, the GEO database was used to validate the reliability of key genes.

**Results:**

Two key genes (ECT2 and COL17A1) were identified, which were highly expressed in PC. The mRNA expression of ECT2 and COL17A1 was associated with DNA methylation and CNV. The cell cycle, proteasome, and pathways in cancer were enriched in the high-COL17A1 and ECT2 groups. The TME results showed that immune scores were decreased in the high-ECT2 group. CeRNA network results showed that there were eleven miRNAs were involved in the regulation of ECT2 and COL17A1. Moreover, pRRophetic analysis showed that 20 chemotherapeutic drugs were associated with ECT2 and COL17A1 expression.

**Conclusions:**

Collectively, ECT2 and COL17A1 may be potential biomarkers for PC, providing a new direction for clinical diagnosis and treatment.

## 1. Introduction

Pancreatic cancer (PC) is an aggressive malignancy with a five-year survival rate of less than 9% [1]. It is expected that in another decade, PC will be the leading contributor to cancer deaths, second only to lung cancer [2, 3]. Poor survival is caused by rapid development of PC, atypical clinical symptoms, and lack of early diagnostic biomarkers. The best treatment for PC is surgical resection, but most patients are diagnosed with metastases that cannot be surgically resected [4]. Chemoradiotherapy, neoadjuvant therapy, radiotherapy, and immunotherapy have some benefits for patients with PC, but its side effects are equally pronounced [5]. Therefore, there is the need for the effective predictive biomarker to provide individualized treatment for patients with PC to improve the quality of survival and prolong survival time. With the development of the molecular biology of tumors, predictive tools based on diagnostic and prognostic-related genes are maturing. These molecular markers may enable a more accurate individualized diagnosis and treatment.

Molecular regulation of tumorigenesis is the most important mechanism of pancreatic carcinogenesis. The development of high-throughput sequencing technology and bioinformatics analysis has confirmed the contribution of key genes in predicting the overall survival and development of PC [6–10]. Song et al. found PRMT1 to be a risk factor for PC development and poor prognosis [7]. Tu et al. found that S100A16 was a potential biomarker for PC affecting patient prognosis [11]. Furthermore, Chen et al. identified a prognostic model consisting of four miRNAs that reliably predicted the prognosis of PC patients and screened for two potential prognostic genes (COL12A1 and COL5A2) [12]. Ye et al. revealed the role of miR-7 as a tumor suppressor and predicted it as a prognostic biomarker in PC [13].

In this study, we screened and validated PC prognosis-related genes using bioinformatics analysis of The Cancer Genome Atlas (TCGA; http://cancergenome.nih.gov/) and the Gene Expression Omnibus (GEO; https://www.ncbi.nlm.nih.gov/geo/) database. R language packages and TCIA (https://tcia.at/home) database were used to explore potential mechanisms and treatment sensitivity of key genes in pancreatic cancer. This may help to reveal the pathogenesis of PC and also provide new biomarkers for the diagnosis and treatment of PC.

## 2. Materials and Methods

### 2.1. Data Collection

The gene expression data and corresponding clinical features of PC and pan-cancer genomic sequencing data were downloaded from TCGA database. And GEO database dataset was used as the test group to verify the results. The advanced search was conducted using the following keywords “pancreatic cancer,” “PAAD,” “survival,” and “Homo sapiens.” “Cell lines” were excluded from the search as an exclusion criterion. The GSE62452 [14] which consisted of 69 tumor tissues and 61 normal tissues was selected and downloaded for subsequent validation analysis. The selected dataset met the following requirements: (1) human pancreatic cancer tissue, (2) tumor and nontumor tissue specimens, and (3) >100 samples. All data were normalized using the robust multiarray average algorithm [15] and log2-transformed for further analysis.

### 2.2. Identification of Differentially Expressed Genes (DEGs)

DEGs between PC and normal samples were identified using the limma package of R [16]. Genes with adjusted *P* values < 0.05 and |log FC| > 2 were identified as DEGs between tumor tissues and normal tissues. Volcano plots and heatmaps were visualized using ggplot2 and pheatmap packages.

### 2.3. Identification of Key Genes

The DEGs were filtered for survival analysis using the Cox and Kaplan-Meier algorithms. Genes with *P* values < 0.001 for all algorithms were further analyzed. Next, a multi-Cox regression analysis was used to analyze the relationship between prognosis and gene expression. The screening criterion for the genes was *P* < 0.001. Finally, the correlation between genes and clinical characteristics was analyzed. The cutoff value for clinical correlation filtering was set at *P* < 0.05. Genes meeting the above three screening conditions were used as key genes for PC for subsequent analysis.

### 2.4. Clinical Value Evaluation

Based on the expression of key genes, PC samples were divided into high- and low-expression groups. Survival and Survminer packages were used to analyze the overall survival (OS) rate and visualize it using Kaplan-Meier plots [17]. To assess the diagnostic value of key genes, the pROC package in R was used to conduct the ROC curve [18].

### 2.5. The DNA Methylation, Mutation, and Copy Number Variation (CNV) Analysis of Key Genes

The DNA methylation and mutation of key genes were analyzed by the cBioPortal database [19] (https://www.cbioportal.org/). The CNV data of PC were obtained from TCGA database, and the CNV of genes was counted by Perl script. The genes with differences in CNV were screened by chi-square test and visualized by chromosome loop diagram.

### 2.6. Gene Set Enrichment Analysis (GSEA)

Based on the expression level of ECT2 and COL17A1, the PC samples were divided into high- and low-expression groups. The reference gene set c5.go.v7.4.symbols.gmt; c2.cp.kegg.v7.4.symbols.gmt were selected, and the clusterProfiler package was used for GSEA [20]. The results with *P* < 0.05 were considered statistically significant.

### 2.7. Estimation of Tumor Microenvironment (TME)

The stromal and immune scores were evaluated using the ESTIMATE algorithm to establish the relationship between ECT2, COL17A1, and TME [21]. The results were presented using the ggpubr package in R. Immune cell identification was performed on the LUAD dataset using the ssGSEA algorithm [22].

### 2.8. Construction of ceRNA Network

The STARBASE database (https://starbase.sysu.edu.cn) contains data on miRNA targets and RNA-RNA interactions. We used the STARBASE database to predict miRNAs of key genes, and miRNAs with a program Num > 2 were selected. Next, coexpression analysis was performed for key genes and miRNAs with the criteria of correlation coefficient > 0.2 and *P* < 0.001. The ceRNA network results were visualized using the Cytoscape software (version 3.8.0).

### 2.9. Immunotherapeutic and Chemotherapeutic Prediction

Immunotherapy sensitivity data for PC were downloaded from TCIA database, and the selected data meet the following criteria: (1) TCGA and (2) PAAD. The results of immunotherapy sensitivity were visualized using the violin plot. Chemotherapy is the primary treatment for terminal PC. The pRRophetic package in R was used to estimate the half-maximal inhibitory concentration (IC50) of the drugs on patients with PC to predict chemotherapy response [23].

### 2.10. Pan-Cancer Analysis and Verification

Transcriptomic and clinical data for pan-cancer were downloaded from TCGA database. The expression and survival of key genes in pan-cancer dataset were analyzed, and the threshold was set at *P* < 0.05. The GSE62452 dataset was used to validate the expression and survival of the key genes in PC.

## 3. Results

### 3.1. Identification of the DEGs and Key Genes

**Figure 1:**
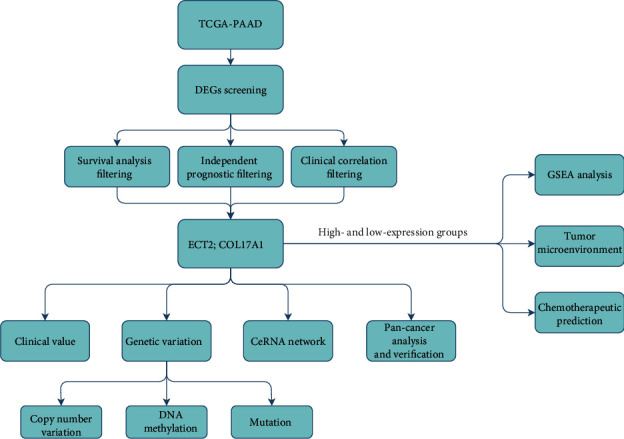
The workflow diagram of this study.

**Figure 2:**
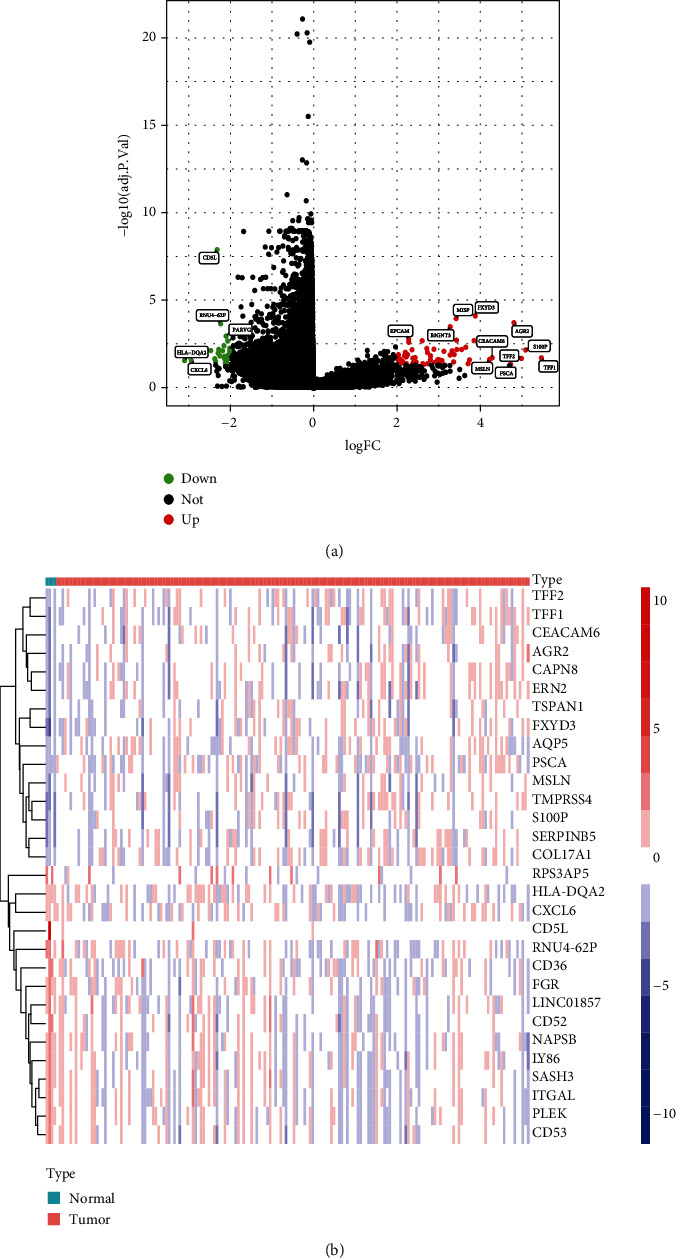
Identification of the differentially expressed genes. (a) The volcano plots of differentially expressed genes. (b) The heatmap of top 30 differentially expressed genes.

The study was conducted as shown in Figure 1. The R package was applied to the dataset for the analysis of differential genes between the PC and normal samples. A total of 241 DEGs were identified, including 81 upregulated and 160 downregulated genes. The results were visualized using a volcano plot (Figure 2(a)), and the top 30 DEGs were illustrated on a heatmap (Figure 2(b)). To screen for key genes in PC, survival analysis filtering was performed for the DEGs. A total of 83 survival related genes were identified (*P* < 0.001), and these genes were then filtered through independent prognostic analysis and clinical correlation filtering. ECT2 and COL17A1 were identified as key genes for further analysis (*P* < 0.05). Detailed results of the screening were presented in Supplementary Table 1.

### 3.2. Evaluation of the Clinical Value of Key Genes

**Figure 3:**
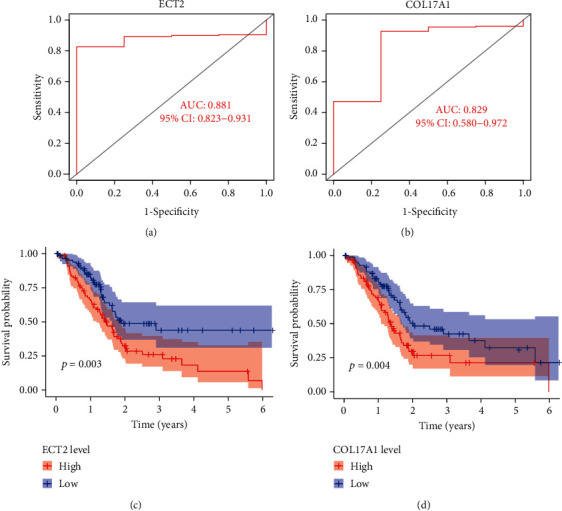
Evaluation of the clinical value of key genes. (a) The ROC curves for ECT2. (b) The ROC curves for COL17A1. (c) The Kaplan-Meier plot for overall survival based on ECT2 expression. (d) The Kaplan-Meier plot for overall survival based on COL17A1 expression.

**Figure 4:**
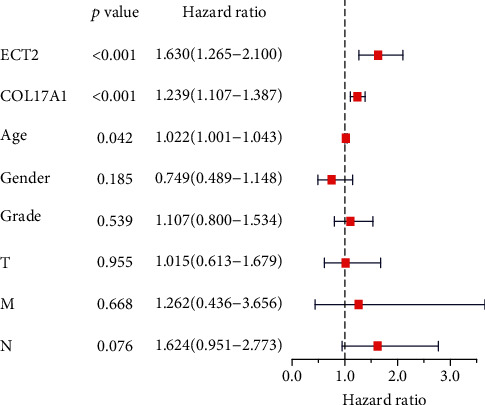
Independent prognostic analysis of key genes in PC.

To assess the clinical value of ECT2 and COL17A1 in PC, ROC and survival analyses were performed. The results showed that the AUC values of ECT2 and COL17A1 were greater than 0.8, and the overall survival (OS) rate decreased with an increase in gene expression (*P* < 0.05) (Figure 3). Next, the results of multivariate Cox regression analysis showed that ECT2 and COL17A1 were high-risk factors for PC and could be independent prognostic factors (*P* < 0.05) (Figure 4). The clinical correlation of key genes was analyzed, and the results showed that the expression of ECT2 and COL17A1 was significantly correlated with tumor grade (*P* < 0.05) (Supplementary figure 1).

### 3.3. DNA Methylation, CNV, and Mutation Analysis of Key Genes

**Figure 5:**
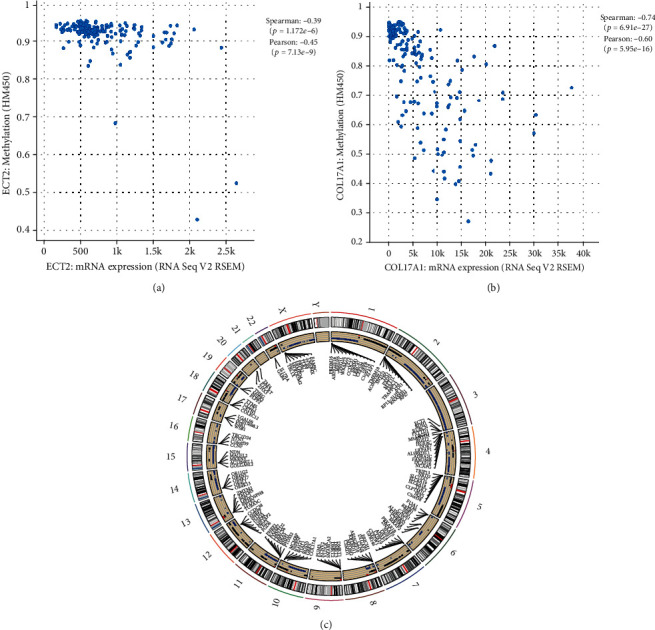
The genetic variation of key genes. (a) Correlation between ECT2 DNA methylation and mRNA expression. (b) Correlation between COL17A1 DNA methylation and mRNA expression. (c) The analysis of CNV on COL17A1 and ECT2.

To investigate the potential mechanisms underlying dysregulation of gene expression in PC, DNA methylation, CNV, and mutation analyses were performed. The cBioPortal database was used to explore the relationship between DNA methylation and mRNA expression levels. DNA methylation of ECT2 and COL17A1 negatively correlated with mRNA expression (*P* < 0.05) (Figures 5(a) and 5(b)). Meanwhile, CNV results showed that the CNVs of ECT2 and COL17A1 were significantly higher than those of the normal group (*P* < 0.05) (Figure 5(c)). As shown in Supplemental figure 2, missense mutations were observed in ECT2 and COL17A1 in PC.

### 3.4. GSEA for Key Genes

**Figure 6:**
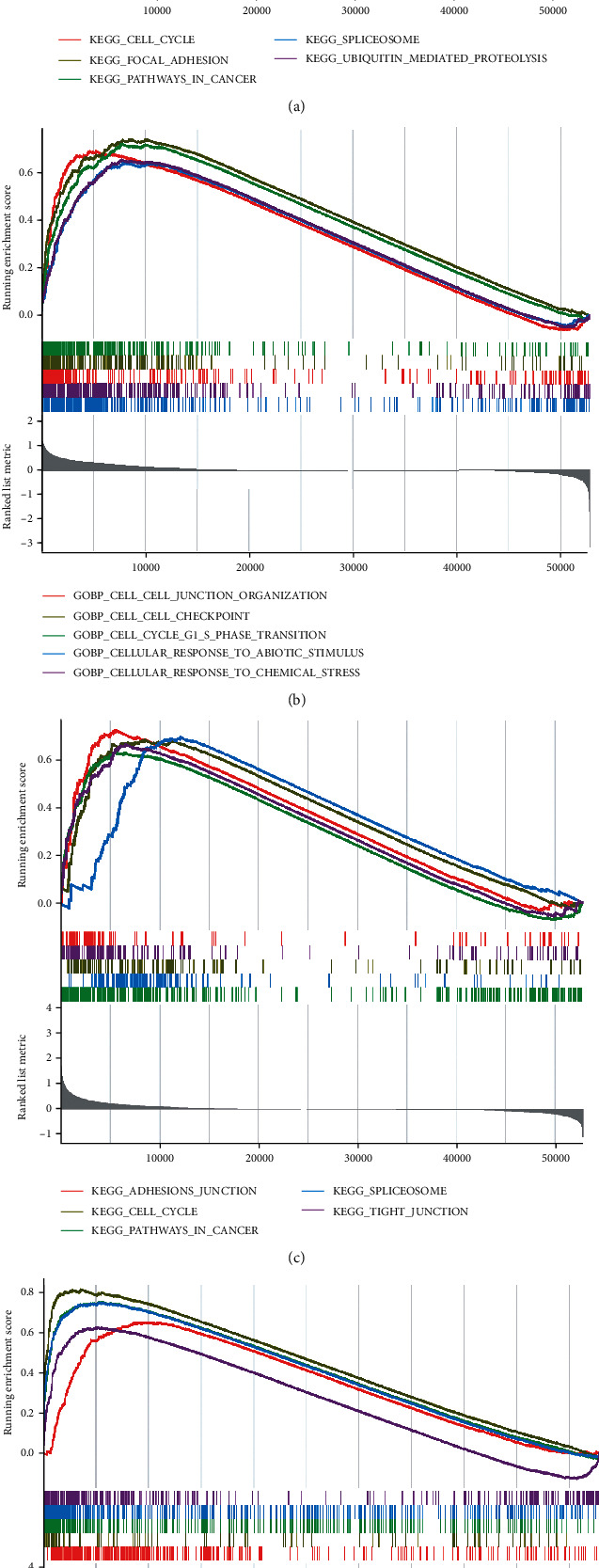
GSEA for key genes. (a) GSEA for the top 5 pathway in the high-ECT2 group; (b) GSEA for the top 5 biological functions in the high-ECT2 group; (c) GSEA for the top 5 pathway in the high-COL17A1 group; (d) GSEA for the top 5 biological functions in the high-COL17A1 group.

To further explore the potential functions of ECT2 and COL17A1 in PC, GSEA of the PC dataset was performed. The dataset was divided into high- and low-expression groups based on ECT2 and COL17A1 expression. The KEGG results showed that the cell cycle, pathways in cancer, and proteasome were greatly enriched in the high-expression group of ECT2 and COL17A1 (*P* < 0.05) (Figures 6(a) and 6(c)). Meanwhile, it was found that the biological functions in the high-ECT2 group were mainly enriched in cell junction and cellular response to stimulus (*P* < 0.05) (Figure 6(b)), and in the high-COL17A1 group, the biological functions were enriched in chromosome segregation and epidermal cell differentiation (*P* < 0.05) (Figure 6(d)).

### 3.5. Estimation of TME

**Figure 7:**
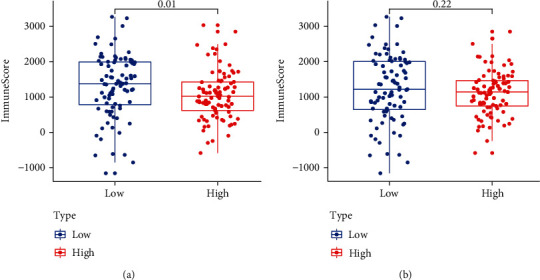
Tumor burden mutation analysis for key genes. (a) Distribution of TMB among high- and low-ECT2 groups. (b) Distribution of TMB among high- and low-COL17A1 groups.

To explore the relationship between the key genes and immune infiltration, the TME analysis was performed on the PC dataset. The results showed that the immune scores were decreased in the high-ECT2 group (*P* < 0.05), while there was no significant difference in COL17A1 expression (Figure 7). The results of immune infiltrating cells showed no significant difference between the PC and normal groups for twenty-one immune cells (Supplemental figure 3), which may be related to the immune escape mechanism of PC.

### 3.6. Construction of ceRNA Network

**Figure 8:**
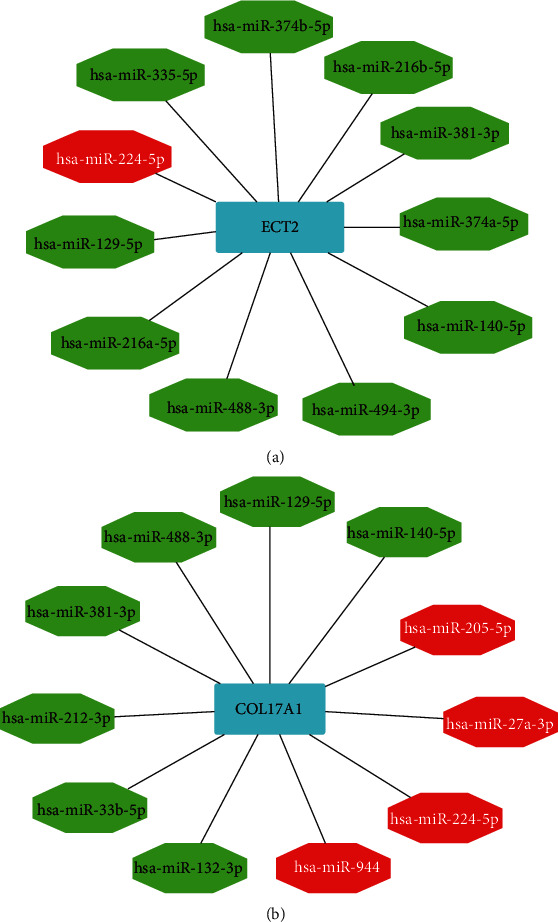
ceRNA network of key genes. (a) The ceRNA network of ECT2. (b) The ceRNA network of COL17A1.

The miRNAs were predicted using the STRABASE database, and ceRNA networks were constructed based on the expression matrix of miRNAs and ECT2 and COL17A1. All the networks are shown in Figure 8. In ECT2, ten miRNAs were found to downregulate ECT2 expression, and one miRNA was found to upregulate ECT2 expression (*P* < 0.001). As for COL17A1, seven miRNAs were identified that could downregulate COL17A1 expression and four miRNAs were identified that could upregulate COL17A1 expression (*P* < 0.001).

### 3.7. Prediction of Potential Drug

**Figure 9:**
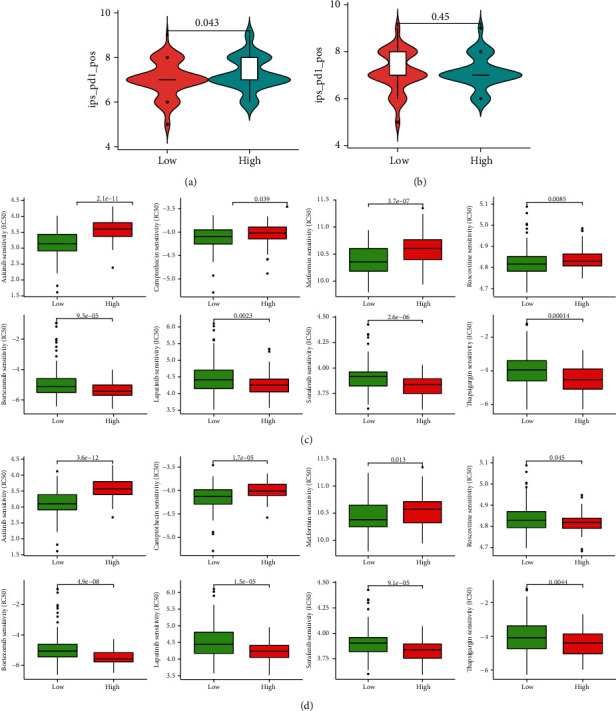
Prediction of potential drug responses. (a) Sensitivity of PD-1 treatment in high- and low-ECT2 groups. (b) Sensitivity of PD-1 treatment in high- and low-COL17A1 groups. (c) Differential chemotherapeutic responses in high- and low-ECT2 groups. (d) Differential chemotherapeutic responses in high- and low-COL17A1 groups.

Immune checkpoint blockade therapy could significantly improve the survival of patients with cancer. The high-ECT2 group showed more sensitivity in response to anti-PD-1 therapy (*P* < 0.05) (Figure 9(a)), while this was not found for COL17A1 (Figure 9(b)). Chemotherapy is an important treatment option for PC. The relationship between ECT2 and COL17A1 and chemotherapeutic agents was further explored. The results showed that the IC50 values of 20 drugs such as bortezomib and rapamycin were correlated with the expression of ECT2 and COL17A1 (*P* < 0.05) (Figure 9, Supplementary figure 4).

### 3.8. Verification of Key Genes

**Figure 10:**
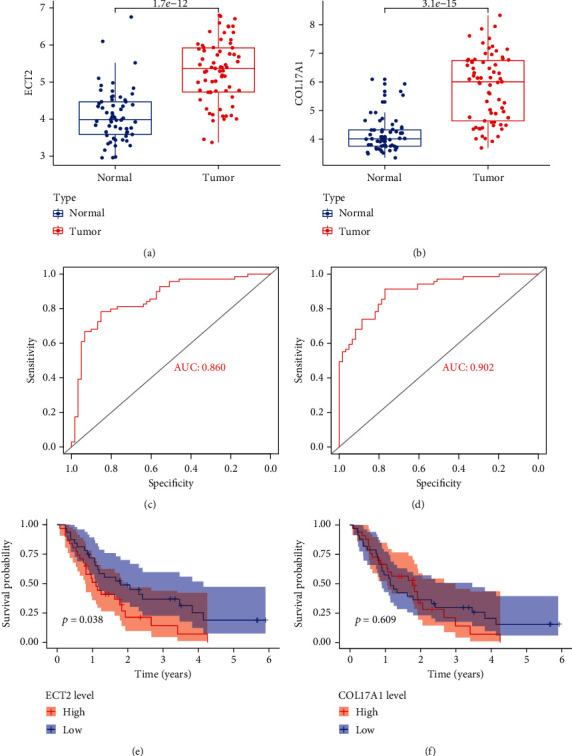
Verification of key genes in GEO dataset. (a) The mRNA expression of ECT2. (b) The mRNA expression of COL17A1. (c) The ROC curves for ECT2. (d) The ROC curves for COL17A1. (e) The Kaplan-Meier curves for overall survival based on ECT2 expression. (f) The Kaplan-Meier curves for overall survival based on COL17A1 expression.

The reliability of ECT2 and COL17A1 was verified by the GEO dataset. The results showed that the expressions of ECT2 and COL17A1 were upregulated in PC (*P* < 0.05) (Figures 10(a) and 10(b)). Meanwhile, the results of ROC and survival analysis showed that ECT2 was significant for the diagnosis and prognosis of PC (*P* < 0.05) (Figures 10(c) and 10(e)). However, in COL17A1, there was no significant difference in overall survival (Figures 10(d) and 10(f)). In addition, the expression and survival of ECT2 and COL17A1 were analyzed in pan-cancer. The results indicated that the expression of ECT2 was different in 19 tumors and correlated with overall survival in 13 tumors (*P* < 0.05) (Supplementary figure 5-6). As for COL17A1, its high expression implied poor prognosis in two tumors and was differentially expressed in 18 tumors (*P* < 0.05) (Supplementary figure 5-6).

## 4. Discussion

In this study, DEGs were screened between normal and PC samples from TCGA database. Then, survival, independent prognostic, and clinical correlation analyses were performed on DEGs, and two key genes (ECT2 and COL17A1) were filtered out. To identify diagnostic and prognostic markers for PC, ROC and survival analyses were conducted on key genes. The results showed that the AUC values of ECT2 and COL17A1 > 0.8 and high expression of ECT2 and COL17A1 were significantly associated with poor PC prognosis. Consistent results were obtained in the GEO database validation; however, there was no significant difference in the survival analysis of COL17A1, which might be due to the small number of specimens. Moreover, the analysis of independent prognostic and clinical correlations demonstrated that ECT2 and COL17A1 could not only be independent prognostic factors of PC but also positively correlated with tumor grade. It was suggested that ECT2 and COL17A1 might be potential biomarkers for the development of PC.

ECT2 is a guanine nucleotide exchange molecule of the Rho family of GTPases that catalyzes the exchange of GDP with GTP to activate downstream signals involved in cell proliferation and rRNA synthesis [24]. ECT2 acts as an oncogene and is involved in the proliferation and metastasis of several tumors. Sun et al. found that ECT2 was involved in the development of esophageal cancer through the RhoA-ERK signal pathway [25]. Zhi et al. found that ECT2 promoted the proliferation of glioma cells by upregulating PTTG1 expression [26]. In this study, the genetic variation in ECT2 was analyzed and found that CNV of ECT2 was significantly increased and DNA methylation was decreased. This might be related to upregulation of ECT2 expression. Furthermore, GSEA results showed that cell cycle, cell junction-related functions, and pathways were significantly enriched in the high-ECT2 group. Cell cycle dysfunction is an important hallmark of tumorigenesis [27]. It was found that inhibition of ECT2 promoted apoptosis of cholangiocarcinoma stem cells and inhibits tumor progression [28]. Therefore, we hypothesized that ECT2 and its related genes act in the cell cycle, which in turn affects the proliferation and metastasis of cancer cells.

XVII collagen is an adhesion protein present in basal epithelial cells, encoded by COL17A1, that affects the growth and migration of cells [29]. COL17A1 is associated with poor prognosis in several tumors [30, 31]. Pulari et al. found that high expression and hypomethylation of COL17A1 were associated with poor prognosis in epithelial carcinoma [32]. Yan et al. found that XVII collagen increased glioma aggressiveness and was associated with glioma recurrence [33]. In this study, DNA methylation of COL17A1 was negatively correlated with mRNA expression and the CNV of COL17A1 was significantly increased. This suggested that the expression of COL17A1 might be affected by DNA methylation and CNV. To explore the mechanism of COL17A1 in PC, the GSEA was performed. The results showed that COL17A1 was associated with epithelial cell development and cell cycle. The transition from a single-layer epithelial structure to a multilayer epithelial structure is an important sign of carcinogenesis [34]. The study identified COL17A1 as an important factor in the formation and maintenance of multilayered epithelial structures [29]. It was suggested that COL17A1 could be involved in development of PC by affecting the differentiation and proliferation of epithelial cells.

The TME is a critical factor influencing tumorigenesis, and tumor cells shape the TME by secreting various cytokines to promote tumor development [35]. A previous study showed that immune cell interactions in the TME could promote tumorigenesis [36]. In this study, the results showed that the immune scores decreased in the high-ECT2 group, while there was no significant difference in COL17A1. This indicated that ECT2 might be involved in the pathogenesis of PC by affecting immune cell infiltration.

It has been shown that miRNA was involved in the progression of PC. Tian et al. found that miRNA-107 regulated TGFBR3 to promote the proliferation and metastasis of PC [37]. It was reported that miRNA-320b could inhibit PC proliferation through FOXM1 [38].To reveal the potential regulatory mechanisms of ECT2 and COL17A1 in PC, the ceRNA network was constructed. Eleven miRNAs regulated the expression of COL17A1, and ten miRNAs downregulated ECT2 expression. This might help enrich the ceRNA regulatory mechanism of ECT2 and COL17A1 and reveal the therapeutic potential of noncoding RNAs in PC.

Recently, immune checkpoint blockade therapy has shown remarkable performance in the treatment of many types of tumors, but little success has been achieved for PC [39]. Some studies have shown that the combination of anti-PD-1 antibody immunotherapy with chemotherapy has been effective in PC [40, 41]. Thus, we made predictions for immune checkpoints and chemotherapeutic drugs. The high-ECT2 group showed more sensitivity in response to anti-PD-1 therapy and 20 chemotherapeutic agents associated with ECT2 and COL17A1. This might provide new ideas for the individualized treatment of PC.

In this study, we investigated the relationship between ECT2, COL17A1, and PC, suggesting that ECT2 and COL17A1 might be used as diagnostic and prognostic markers for PC. However, there are many limitations to our study. Due to the complexity of the data, we were unable to consider the effect of ethnicity, age, gender, and tumor stage on the results. Then, we have found that ECT2 and COL17A1 were involved in pancreatic carcinogenesis, but more biological experiments were needed to verify the mechanism. In addition, bioinformatic analysis alone could not fully explain the prognostic role of ECT2 and COL17A1 in PC, and we will further investigate it in a large-scale population.

In conclusion, we found that ECT2 and COL17A1 were associated with the development and prognosis of PC. ECT2 and COL17A1 might serve as potential biomarkers for PC, providing additional ideas for clinical diagnosis and individualized treatment. However, further experiments are required to investigate the clinical value of ECT2 and COL17A1.

## Data Availability

All data generated or analyzed during this study are available in the GEO (GSE62452) (https://www.ncbi.nlm.nih.gov/geo/), TCGA (http://cancergenome.nih.gov/), and TCIA (https://tcia.at/home) database repository.

## Supplementary Materials

Supplementary Materials Supplemental Table 1: correlation between key genes and clinical traits. Supplemental Figure 1: correlation with ECT2, COL17A1, and tumor grade. Supplemental Figure 2: the mutation analysis of ECT2 and COL17A1. Supplemental Figure 3: the difference of immune cells between normal group and tumor group. Supplemental Figure 4: differential chemotherapeutic responses (A) in high- and low-ECT2 groups and (B) in high- and low-COL17A1 groups. Supplemental Figure 5: ECT2 and COL17A1 levels in different human cancer types (^∗^*P* < 0.001). Supplemental Figure 6: Kaplan-Meier survival curves of ECT2 and COL17A1 expression in different tumor types.
