# Supplementary material for: Integrated Analysis of ECT2 and COL17A1 as Potential Biomarkers for Pancreatic Cancer
Source: Dis Markers. 2022 Jun 8;2022:9453549. doi: 10.1155/2022/9453549 (PMC9200569; doi:10.1155/2022/9453549)
Supplement: Supplementary Materials — Supplemental Table 1: correlation between key genes and clinical traits. Supplemental Figure 1: correlation with ECT2, COL17A1, and tumor grade. Supplemental Figure 2: the mutation analysis of ECT2 and COL17A1. Supplemental Figure 3: the difference of immune cells between normal group and tumor group. Supplemental Figure 4: differential chemotherapeutic responses (A) in high- and low-ECT2 groups and (B) in high- and low-COL17A1 groups. Supplemental Figure 5: ECT2 and COL17A1 levels in different human cancer types (∗P < 0.001). Supplemental Figure 6: Kaplan-Meier survival curves of ECT2 and COL17A1 expression in different tumor types. [file 9453549.f1.zip › supplemental figure (1).pdf]

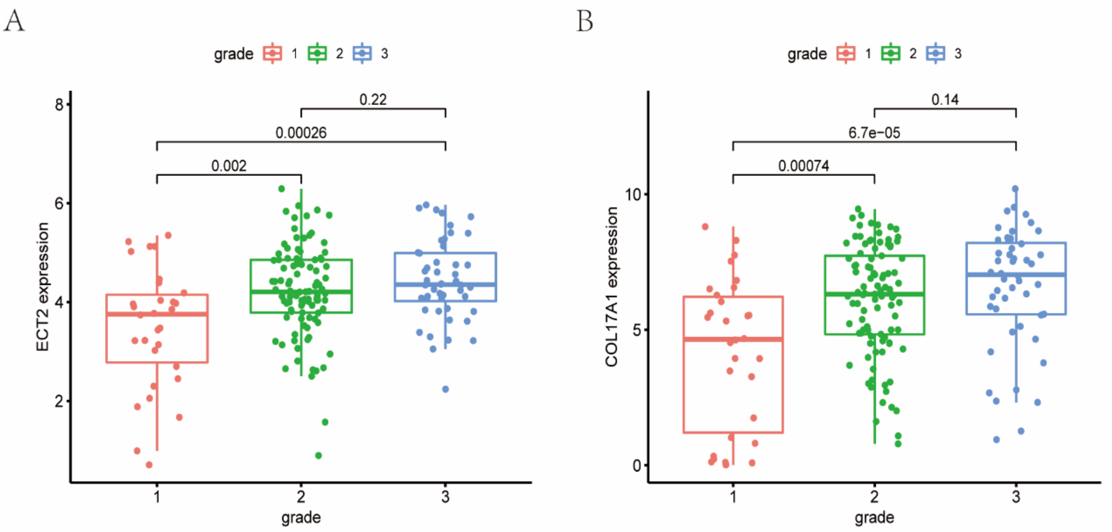

Supplemental figure 1. Correlation with ECT2, COL17A1 and tumor grade.

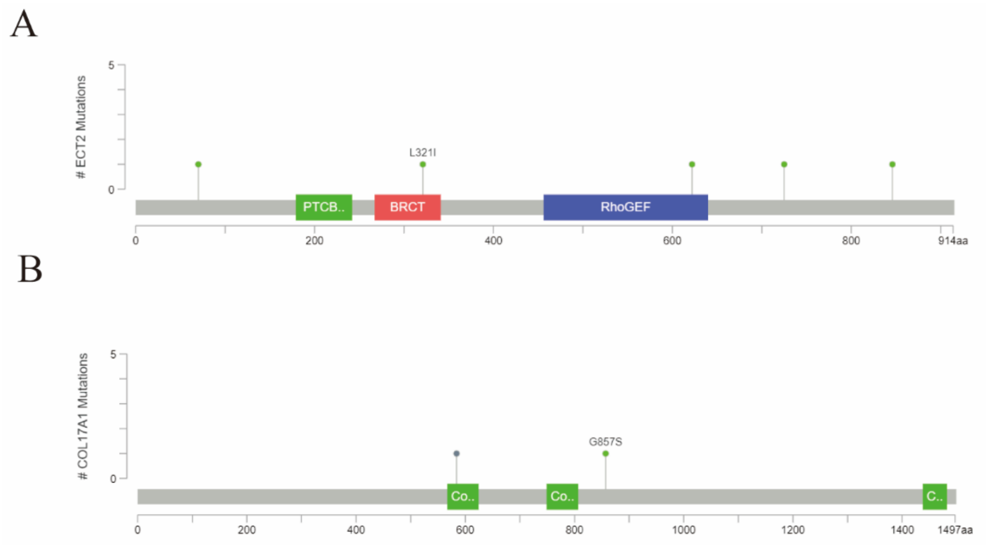

Supplemental figure 2. The mutation analysis of ECT2 and COL17A1.

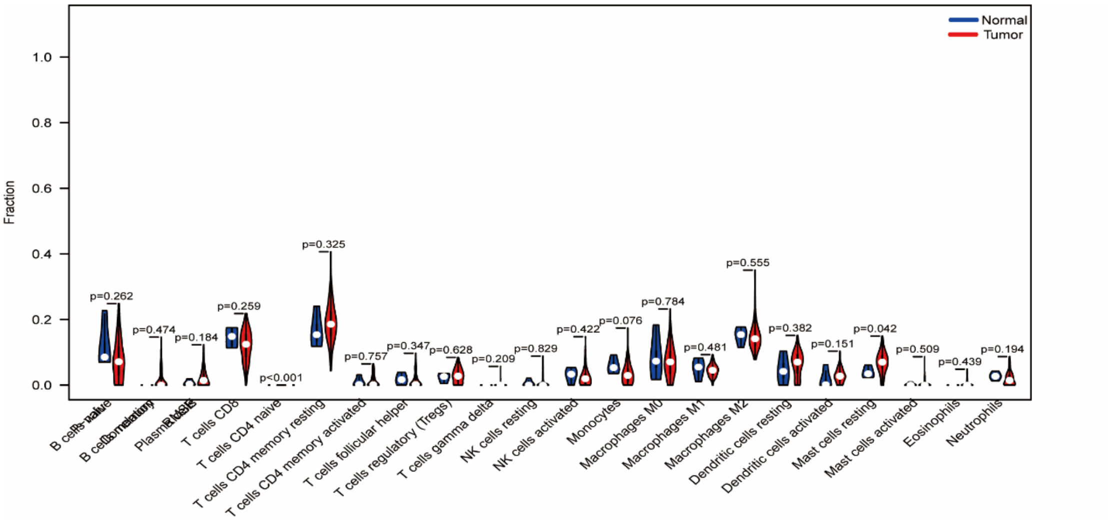

Supplemental figure 3. The difference of immune cells between normal group and tumor group.

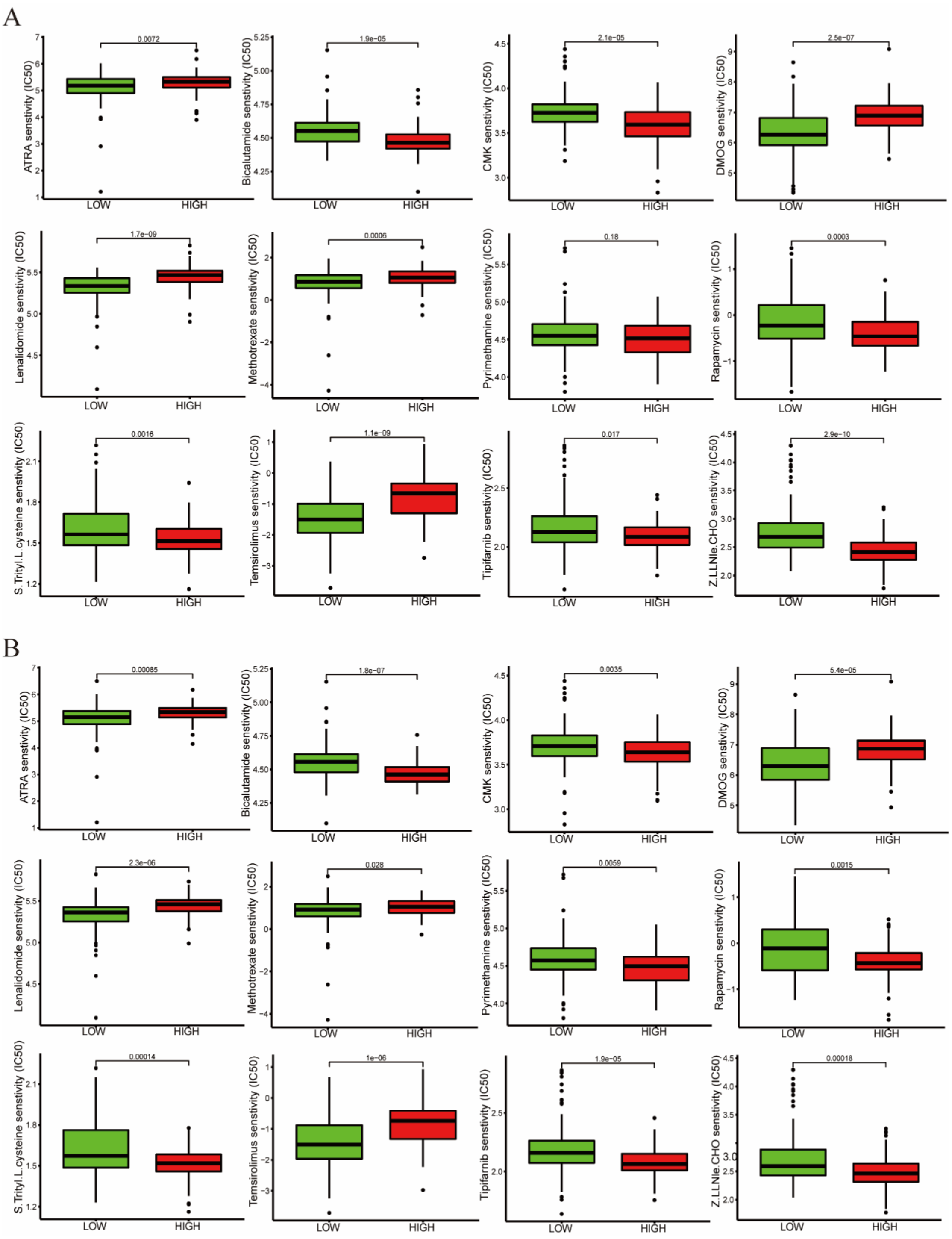

Supplemental figure 4. Differential chemotherapeutic responses (A)in high- and low-ECT2 group and (B)in high- and low-COL17A1 group.

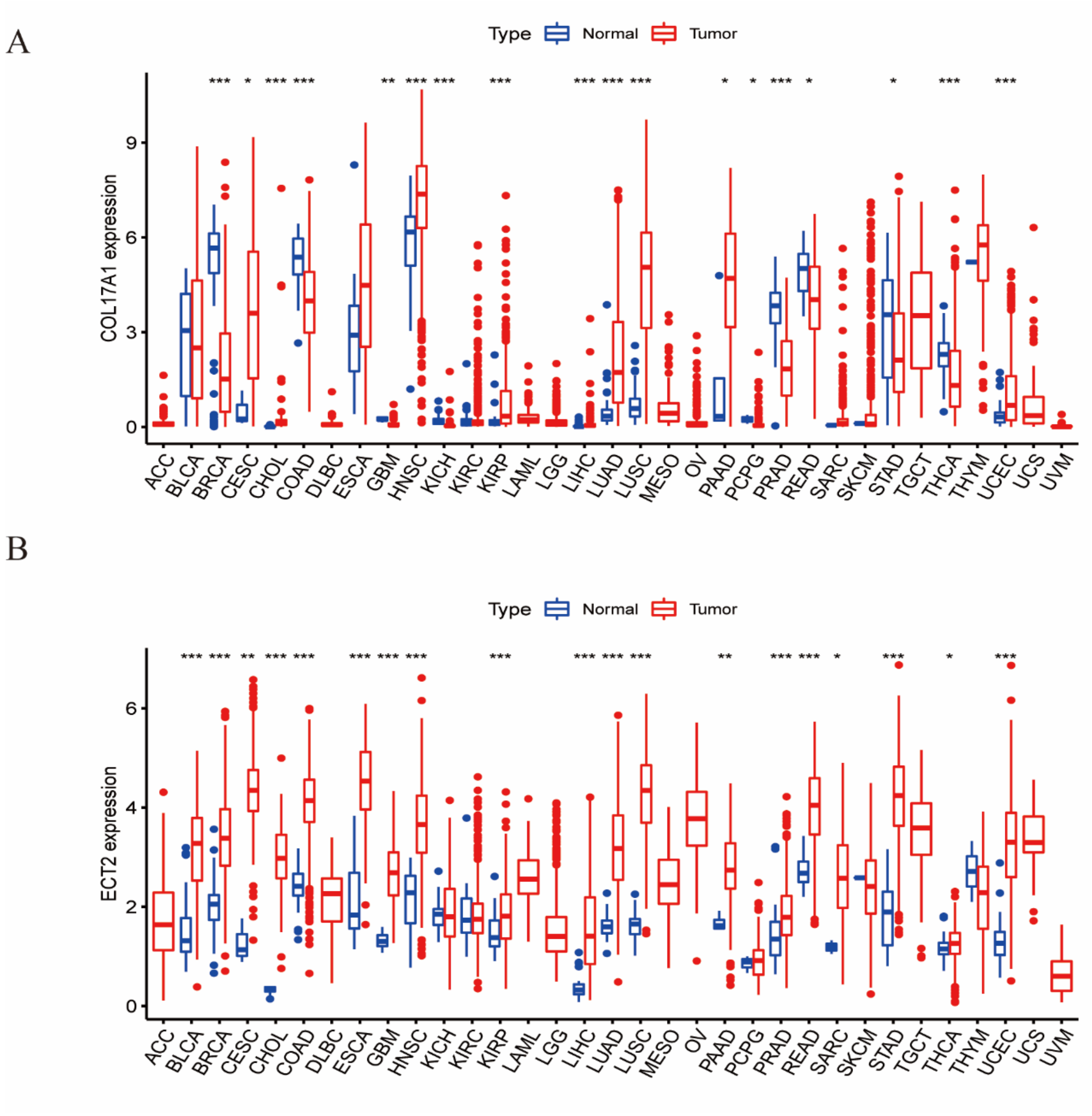

Supplemental figure 5. ECT2 and COL17A1 levels in different human cancer types. (* P<0.05; ** P<0.01; ***P<0.001)

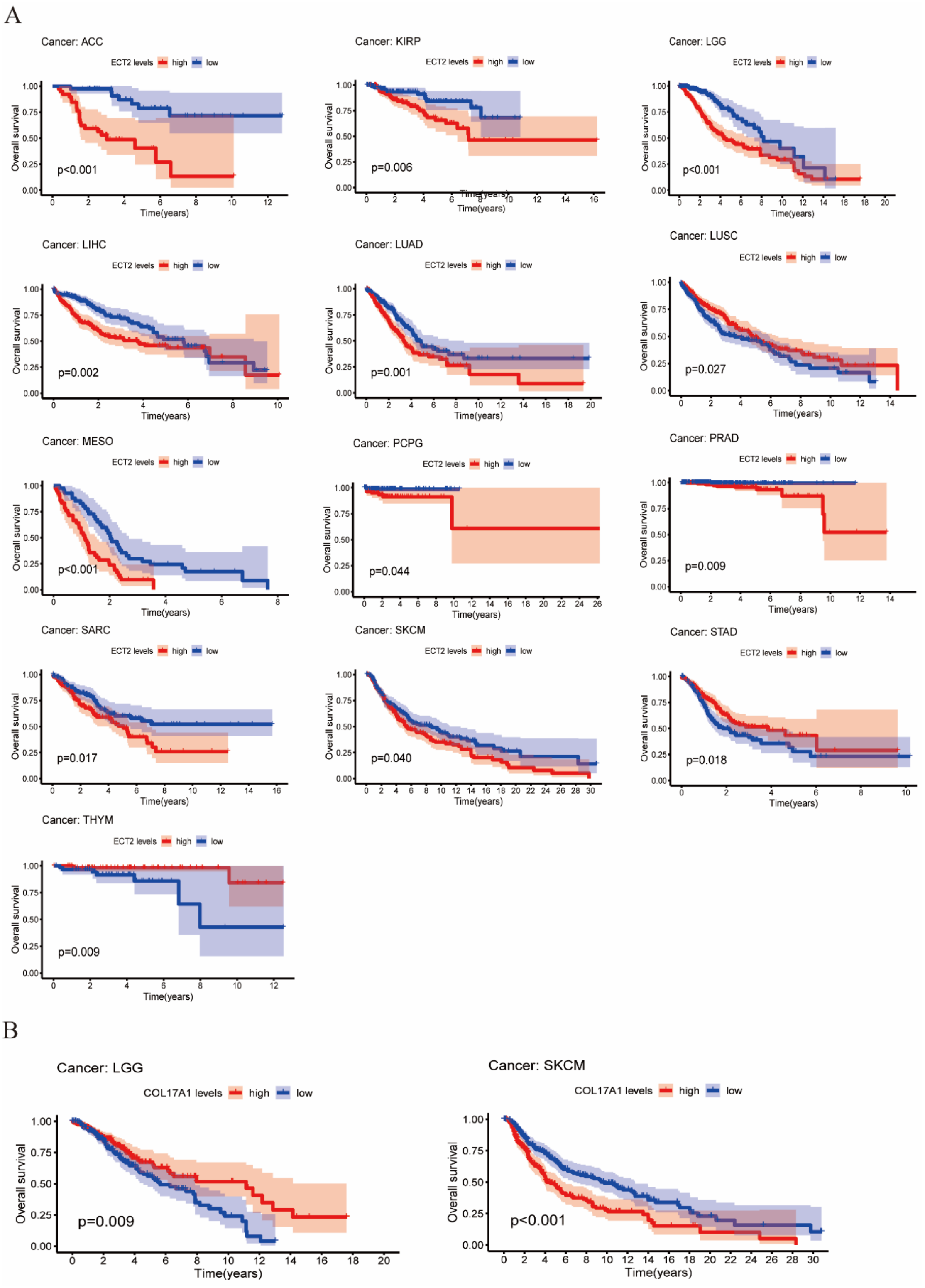

Supplemental figure 6. Kaplan-Meier survival curves of ECT2 and COL17A1 expression in different tumor types.

Supplemental table 1. Correlation between key genes and clinical traits

id	age	gender	grade	stage	T	M	N	SigNum
ECT2	0.976972	0.719997	0.002668	0.023579	0.004335	0.294379	0.146965	2
COL17A1	0.478024	0.087276	0.000704	0.041791	0.006129	0.98543	0.796409	2
GPRC5A	0.656221	0.505035	0.00071	0.052023	0.037475	0.537153	0.395361	1
MET	0.028142	0.512863	0.000198	0.122065	0.019826	0.115335	0.714744	1
MKI67	0.237822	0.749875	0.006085	0.295495	0.069576	0.851103	0.574878	1
ANLN	0.904467	0.911337	0.00084	0.260378	0.133493	0.553614	0.228852	1
LIPH	0.187779	0.659333	0.003246	0.028452	0.049118	0.666741	0.755088	1
AHNAK2	0.723352	0.575607	1.84E-05	0.136301	0.376821	0.143911	0.459567	1
LAMA3	0.499002	0.787167	0.000285	0.061434	0.285677	0.455152	0.12667	1
ITGA2	0.05816	0.710886	0.000519	0.060943	0.015888	0.14222	0.243456	1
LAMB3	0.861306	0.108225	0.002771	0.120107	0.334335	0.537697	0.207032	1
SERPINB5	0.52937	0.730292	0.00013	0.289587	0.127295	0.851306	0.864753	1
ITGA3	0.089126	0.505035	6.20E-05	0.192009	0.220892	0.688311	0.912167	1
ARNTL2	0.041015	0.976879	0.000942	0.250904	0.121869	0.150331	0.486752	1
DSG3	0.298728	0.425	0.000382	0.052443	0.092188	0.441485	0.273272	1
CENPF	0.560656	0.933139	0.009354	0.289786	0.134205	0.507718	0.423803	1
ITGB6	0.212289	0.728001	0.000423	0.099831	0.175283	0.869383	0.152448	1
ANO1	0.412861	0.947703	0.001007	0.522579	0.608675	0.116614	0.952916	1
CDH3	0.878049	0.630877	1.51E-05	0.043182	0.029355	0.453904	0.556101	1
LMO7	0.356436	0.253244	0.002886	0.091964	0.032144	0.326227	0.611218	1
IL1RAP	0.026861	0.210448	0.000165	0.105827	0.223582	0.045042	0.122492	1
ASPM	0.581302	0.984179	0.002106	0.081306	0.018787	0.440032	0.419459	1
MYOF	0.018914	0.77312	1.06E-05	0.083942	0.034449	0.023175	0.178618	1
TOP2A	0.899655	0.397227	0.022973	0.334904	0.121964	0.768524	0.47945	0
KRT7	0.229473	0.067411	0.291948	0.134357	0.202609	0.755824	0.70289	0
KRT19	0.979395	0.301754	0.036159	0.885081	0.916245	0.878826	0.83324	0
DCBLD2	0.040418	0.32819	0.013966	0.465848	0.516829	0.018504	0.176563	0
NT5E	0.023131	0.817834	0.113257	0.70644	0.220943	0.037675	0.283837	0
