# Supplementary material for: Integrated Analysis of ECT2 and COL17A1 as Potential Biomarkers for Pancreatic Cancer
Source: Dis Markers. 2022 Jun 8;2022:9453549. doi: 10.1155/2022/9453549 (PMC9200569; doi:10.1155/2022/9453549)
Supplement: Supplementary Materials — Supplemental Table 1: correlation between key genes and clinical traits. Supplemental Figure 1: correlation with ECT2, COL17A1, and tumor grade. Supplemental Figure 2: the mutation analysis of ECT2 and COL17A1. Supplemental Figure 3: the difference of immune cells between normal group and tumor group. Supplemental Figure 4: differential chemotherapeutic responses (A) in high- and low-ECT2 groups and (B) in high- and low-COL17A1 groups. Supplemental Figure 5: ECT2 and COL17A1 levels in different human cancer types (∗P < 0.001). Supplemental Figure 6: Kaplan-Meier survival curves of ECT2 and COL17A1 expression in different tumor types. [file 9453549.f1.zip › supplemental table 1.docx]

| id | age | gender | grade | stage | T | M | N | SigNum |
| --- | --- | --- | --- | --- | --- | --- | --- | --- |
| ECT2 | 0.976972 | 0.719997 | 0.002668 | 0.023579 | 0.004335 | 0.294379 | 0.146965 | 2 |
| COL17A1 | 0.478024 | 0.087276 | 0.000704 | 0.041791 | 0.006129 | 0.98543 | 0.796409 | 2 |
| GPRC5A | 0.656221 | 0.505035 | 0.00071 | 0.052023 | 0.037475 | 0.537153 | 0.395361 | 1 |
| MET | 0.028142 | 0.512863 | 0.000198 | 0.122065 | 0.019826 | 0.115335 | 0.714744 | 1 |
| MKI67 | 0.237822 | 0.749875 | 0.006085 | 0.295495 | 0.069576 | 0.851103 | 0.574878 | 1 |
| ANLN | 0.904467 | 0.911337 | 0.00084 | 0.260378 | 0.133493 | 0.553614 | 0.228852 | 1 |
| LIPH | 0.187779 | 0.659333 | 0.003246 | 0.028452 | 0.049118 | 0.666741 | 0.755088 | 1 |
| AHNAK2 | 0.723352 | 0.575607 | 1.84E-05 | 0.136301 | 0.376821 | 0.143911 | 0.459567 | 1 |
| LAMA3 | 0.499002 | 0.787167 | 0.000285 | 0.061434 | 0.285677 | 0.455152 | 0.12667 | 1 |
| ITGA2 | 0.05816 | 0.710886 | 0.000519 | 0.060943 | 0.015888 | 0.14222 | 0.243456 | 1 |
| LAMB3 | 0.861306 | 0.108225 | 0.002771 | 0.120107 | 0.334335 | 0.537697 | 0.207032 | 1 |
| SERPINB5 | 0.52937 | 0.730292 | 0.00013 | 0.289587 | 0.127295 | 0.851306 | 0.864753 | 1 |
| ITGA3 | 0.089126 | 0.505035 | 6.20E-05 | 0.192009 | 0.220892 | 0.688311 | 0.912167 | 1 |
| ARNTL2 | 0.041015 | 0.976879 | 0.000942 | 0.250904 | 0.121869 | 0.150331 | 0.486752 | 1 |
| DSG3 | 0.298728 | 0.425 | 0.000382 | 0.052443 | 0.092188 | 0.441485 | 0.273272 | 1 |
| CENPF | 0.560656 | 0.933139 | 0.009354 | 0.289786 | 0.134205 | 0.507718 | 0.423803 | 1 |
| ITGB6 | 0.212289 | 0.728001 | 0.000423 | 0.099831 | 0.175283 | 0.869383 | 0.152448 | 1 |
| ANO1 | 0.412861 | 0.947703 | 0.001007 | 0.522579 | 0.608675 | 0.116614 | 0.952916 | 1 |
| CDH3 | 0.878049 | 0.630877 | 1.51E-05 | 0.043182 | 0.029355 | 0.453904 | 0.556101 | 1 |
| LMO7 | 0.356436 | 0.253244 | 0.002886 | 0.091964 | 0.032144 | 0.326227 | 0.611218 | 1 |
| IL1RAP | 0.026861 | 0.210448 | 0.000165 | 0.105827 | 0.223582 | 0.045042 | 0.122492 | 1 |
| ASPM | 0.581302 | 0.984179 | 0.002106 | 0.081306 | 0.018787 | 0.440032 | 0.419459 | 1 |
| MYOF | 0.018914 | 0.77312 | 1.06E-05 | 0.083942 | 0.034449 | 0.023175 | 0.178618 | 1 |
| TOP2A | 0.899655 | 0.397227 | 0.022973 | 0.334904 | 0.121964 | 0.768524 | 0.47945 | 0 |
| KRT7 | 0.229473 | 0.067411 | 0.291948 | 0.134357 | 0.202609 | 0.755824 | 0.70289 | 0 |
| KRT19 | 0.979395 | 0.301754 | 0.036159 | 0.885081 | 0.916245 | 0.878826 | 0.83324 | 0 |
| DCBLD2 | 0.040418 | 0.32819 | 0.013966 | 0.465848 | 0.516829 | 0.018504 | 0.176563 | 0 |
| NT5E | 0.023131 | 0.817834 | 0.113257 | 0.70644 | 0.220943 | 0.037675 | 0.283837 | 0 |

Correlation between key genes and clinical traits
